# Effect of Poor Nutritional Status and Comorbidities on the Occurrence and Outcome of Pneumonia in Elderly Adults

**DOI:** 10.3389/fmed.2021.719530

**Published:** 2021-10-12

**Authors:** Bo Chen, Wen Liu, Yanbing Chen, Quan She, Min Li, HongYe Zhao, Weihong Zhao, Zhihang Peng, Jianqing Wu

**Affiliations:** ^1^Department of Geriatrics, Jiangsu Provincial Key Laboratory of Geriatrics, First Affiliated Hospital, Nanjing Medical University, Nanjing, China; ^2^First Clinical Medical College of Nanjing Medical University, Nanjing, China; ^3^Department of General Practice, The First People's Hospital of Lianyungang, Lianyungang Clinical College of Nanjing Medical University, Lianyungang, China; ^4^Department of Epidemiology and Health Statistics, School of Public Health, Nanjing Medical University, Nanjing, China

**Keywords:** pneumonia, poor nutritional status, comorbidity, outcome, elderly

## Abstract

**Background:** Malnutrition and comorbidity are two common geriatric syndromes. The pathology of pneumonia is multifactorial, making its diagnosis and management a great challenge. Hospital-acquired pneumonia (HAP) and community-acquired pneumonia (CAP) are two main types of pneumonia. However, the effect of geriatric syndromes on pneumonia and its prognosis have not been clearly explored.

**Methods:** We collected the relevant electronic data of inpatients aged over 65 years and diagnosed with pneumonia in the Geriatrics Department Building of the First Affiliated Hospital with Nanjing Medical University between December 2018 and December 2019, and further divided them into HAP group and CAP group. The correlations of age, age-adjusted Charlson Comorbidity Index (aCCI), basic diseases and nutritional indexes (i.e., albumin, electrolyte, hemoglobin) with pneumonia and prognosis were analyzed. We analyzed the associations between infection prognosis/infection level and age, nutritional status, aCCI and underlying diseases, using linear regression model. The box plot was applied to present infection outcome, and the nomogram was built for predicting infection outcomes. We utilized the heat map to show the associations between nutritional status and infection level/outcome in all infected patients, HAP, and CAP.

**Results:** The final study comprised samples of 669 pneumonia patients divided into HAP group (*n* = 517) and CAP group (*n* = 152). In all patients, the infection outcome was negatively correlated with age (*P* = 0.013). The level of albumin was negatively correlated with infection prognosis in all patients (*P* = 0.03), and negatively correlated with neutrophil count and CRP (*P* = 0.008, *P* < 0.001). ACCI was positively correlated with CRP (*P* = 0.003). The prognosis was negatively associated with age and albumin level. In the patients with basic dementia/Alzheimer's disease and chronic obstructive pulmonary disease/asthma, the prognosis was worse.

**Conclusion:** There was a correlation between poor nutritional status-related indexes and inflammatory indexes. A poor nutritional status might predict a high risk of pneumonia in elderly adults. Advanced age and comorbidities were risk factors for the occurrence and poor prognosis of pneumonia. Therefore, comorbidities should be well-treated in the elderly with pneumonia.

## Introduction

Around the world, people are living longer and it is estimated that by 2050, the proportion of the world's population over the age of 60 will nearly double, and the total number of elderly people aged 65 or older will reach 3.200 million (1, 2). The elderly, especially those aged over 80 years, are at high risk of chronic diseases. The mortality due to chronic diseases in China is 533/100,000, accounting for 86.6% of the total deaths, far higher than the global average level (3). Older people are more likely to suffer from comorbidities. The concept of the comorbidity (4) was first proposed by Professor Feinstein in the United States in 1970 (5). Comorbidities can be interrelated or concurrent (6). Especially in cases of infectious diseases, comorbidities act as a catalyst in the occurrence and progression of the disease.

Poor nutritional status is a poor health condition that mainly reflects an inadequate supply of nutrients to meet the physical needs of the body. With the increase of age, related psychological and physiological changes, such as comorbidity, loss of appetite, poor oral health, loss of autonomous eating ability, cognitive impairment, reduced organ function and loss of function, lead to insufficient food intake, as well as absorption and metabolism disorders in the elderly (7–9). As a result, poor nutritional status is particularly common among the elderly. A study using the Mini Nutritional Assessment (MNA) found that the prevalence of poor nutritional status was 20% worldwide, 5.8% in the community and 50.5% in clinical and long-term care facilities (10). Another study showed that 25% of older people in communities were at risk of poor nutritional status and 5% were malnourished, and confirmed that poor nutritional status was associated with increased long-term mortality over 10 years (11, 12). Under the condition of poor nutritional status, the body's resistance decreases and it is prone to infection-related diseases. Under the condition of infection, the body's higher nutrient consumption further leads to the aggravation of poor nutritional status, and the two are mutually causal.

Infectious diseases, especially pneumonia, are the most common cause of hospitalization and death among the elderly patients (13, 14). Compared with that in normal adults, the pneumonia in the elderly, especially those with the advanced ages, is more difficult to be diagnosed and treated. Because the clinical symptoms and the examination results are not typical in the elderly, the diagnosis is more likely to miss, false and delay. Infection prolongates the course and worsens the prognosis of disease. Most of the infected elderly people are accompanied by poor nutritional status and underlying diseases, suggesting that comorbidities are potentially related to the occurrence of infection.

Based on elderly patients with clinical symptoms not obvious, inspection and test results not typical, are more likely to happen in diagnosis and misdiagnosis, delay condition, once the merger or poor nutritional status and a variety of basic diseases increased the risk of elderly patients with lung infection, aggravating illness, extend the course of the disease, diagnosis and treatment for the disease cause more trouble, lead to irreversible consequences. At present, the diagnosis and treatment of pneumonia in elderly patients is still a great challenge. Therefore, based on the clinical data of 669 patients with confirmed pneumonia, this study aimed to reveal the correlation between pneumonia in the elderly and nutrition level, comorbidities and different age groups, so as to provide a reference for early active prevention and treatment of risk factors, effective reduction or avoidance of infection, and clinical comprehensive treatment for elderly patients with pneumonia.

## Methods

### Study Design and Participants

Patients with pneumonia in the Department of Geriatrics, The First Affiliated Hospital of Nanjing Medical University from December 2018 to December 2019 were consecutively collected. Inclusion criteria: (1) age ≥65 years; (2) Clear diagnosis of pneumonia. Exclusion criteria: (1) age <65; (2) patients with hormone and immunosuppressive drugs; (3) patients undergoing radiotherapy and chemotherapy. This study was approved by the Ethics Committee of the First Affiliated Hospital of Nanjing Medical University. In this retrospective study, we screened inpatients aged over 65 years and diagnosed with pneumonia through the electronic medical record system, and further divided them into HAP group and CAP group. HAP diagnostic criteria strictly followed the international ERS/ESICM/ESCMID/ALAT guidelines for the management of HAP and ventilator-associated pneumonia (VAP) and the Chinese Guidelines for the Diagnosis and Treatment of HAP/VAP in Adults (2018 Edition) (15, 16). The diagnosis of CAP was established according the Chinese Guidelines for Adult CAP (2016 Edition) (17).

### Data Collection

The following indicators were collected in this study: white blood cell count (WBC, normal range, 4.0–10.0 × 10^9^/L), neutrophil count (normal range, 1.80–6.30 × 10^9^/L), neutrophil percentage (normal range, 40–75%), C-reactive protein (CRP, normal range, 800–8,000 ug/L) and procalcitonin (PCT, normal range, <0.5 ug/L) as inflammatory markers; albumin (normal range, 3.5–5.0 g/dL), electrolyte (calcium, Ca, normal range 1.10–1.34 mmol/L; potassium, K, normal range 3.5–5.5 mmol/L; sodium, Na, normal range 135–145 mmol/L; chlorine, Cl, normal range 95–105 mmol/L), hemoglobin (Hb, 12.0–16.5 g/dL for males, 11.0–15.0 g/dL for females), lymphocyte (normal range, 20–40%) as nutrient related indicators. Blood tests were all taken before anti-infection treatment of pneumonia. And all patients were treated according to guidelines for anti-infective therapy. The outcome of infection was divided into two groups according to whether the infection resulted in death, the death group and the non-death group. The non-death group was further divided into four groups based on the duration of pneumonia control: ≤7 days, 8–14 days, 15–21 days, ≥22 days. Age-adjusted Charlson Comorbidity Index (aCCI) is more widely used than CCI in scoring comorbidities. It incorporates the age of the patient as a correction variable of the final score. It quantifies comorbidities based on the number and severity of diseases a patient can be used to predict the risk of death from the disease (18–20).

The above information was obtained from the Medical Record System of the First Affiliated Hospital of Nanjing Medical University.

### Statistical Analyses

Pearson or Spearman correlation analysis was used to calculate the correlation coefficient. Quantitative data in normal distribution between groups was compared by ANOVA. Kruskal-Wallis test was used to compare the quantitative data not in normal distribution. Pearson chi-square test or Fisher's exact probability method was used to compare the classification data between groups. Linear regression model was used to analyze the association between infection prognosis/infection level and age, nutritional status, aCCI and underlying diseases. Logistic regression model was used for prediction, and stepwise screening strategy was used for basic diseases, nutritional indexes and inflammatory indexes. We also calculated AUC and draw ROC curve and Nomogram for risk assessment.

All analyses were conducted using the Stata (13.0) and R (3.6.1), and *P* < 0.05 was set as statistically significant.

## Results

### Baseline Clinical Characteristics

Among the 669 patients, 486 (72.65%) were men with the average age of 84.87 ± 8.49 years. Their average aCCI was 6.34 ± 1.97, and 42.60% received anti-infection treatment of ≤7 days, 28.91% of 8–14 days, 13.14% of 15–21 days, 5.81% of ≥22 days and 9.54% of death. There was no significant difference in sex, age, aCCI, and most underlying diseases between the two groups (*P* > 0.05). Patients with myocardial infarction had significantly escalated risks of negative outcomes compared with those without (*P* = 0.031). Chronic obstructive pulmonary disease is another important factor related to infection outcome (*P* = 0.024) (Table 1).

**Table 1:** Baseline infection outcome.

		**≤7 days**	**8–14 days**	**15–21 days**	**≥22 days**	**Death**	**χ^**2**^*/F***	***P***
Gender	Male	165 (64.71)	144 (68.9)	61 (64.21)	29 (69.05)	48 (70.59)	1.743	0.783
	Female	90 (35.29)	65 (31.1)	34 (35.79)	13 (30.95)	20 (29.41)		
Age (year)	65–79	71 (27.84)	42 (20.1)	27 (28.42)	12 (28.57)	21 (30.88)	15.257	0.054
	80–89	100 (39.22)	86 (41.15)	31 (32.63)	16 (38.1)	35 (51.47)		
	≥90	84 (32.94)	81 (38.76)	37 (38.95)	14 (33.33)	12 (17.65)		
Albumin (g/L)	<35	171 (67.06)	132 (63.16)	73 (76.84)	32 (76.19)	47 (69.12)	7.104	0.130
	≥35	84 (32.94)	77 (36.84)	22 (23.16)	10 (23.81)	21 (30.88)		
Lymphocyte count (10∧9/L)	<1.1	127 (49.8)	95 (45.45)	49 (51.58)	28 (66.67)	30 (44.12)	7.255	0.123
	≥1.1	128 (50.2)	114 (54.55)	46 (48.42)	14 (33.33)	38 (55.88)		
Hb (g/L)	<130	193 (75.69)	162 (77.51)	69 (72.63)	36 (85.71)	53 (77.94)	3.070	0.546
	≥130	62 (24.31)	47 (22.49)	26 (27.37)	6 (14.29)	15 (22.06)		
aCCI	≤3	16 (6.27)	10 (4.78)	3 (3.16)	0 (0)	4 (5.88)	5.647	0.227
	4	31 (12.16)	25 (11.96)	9 (9.47)	6 (14.29)	6 (8.82)		
	5	45 (17.65)	45 (21.53)	22 (23.16)	9 (21.43)	11 (16.18)		
	≥6	163 (63.92)	129 (61.72)		27 (64.29)	47 (69.12)		
Myocardial infarction	NO	227 (89.02)	199 (95.22)	86 (90.53)	35 (83.33)	58 (85.29)	10.638	**0.031**
	Yes	28 (10.98)	10 (4.78)	9 (9.47)	7 (16.67)	10 (14.71)		
peripheral vascular disease	NO	225 (88.24)	194 (92.82)	88 (92.63)	35 (83.33)	64 (94.12)	6.928	0.140
	Yes	30 (11.76)	15 (7.18)	7 (7.37)	7 (16.67)	4 (5.88)		
cerebrovascular disease/TIA	NO	100 (39.22)	87 (41.63)	27 (28.42)	19 (45.24)	30 (44.12)	6.470	0.167
	Yes	155 (60.78)	122 (58.37)	68 (71.58)	23 (54.76)	38 (55.88)		
Dementia/Alzheimer's disease	NO	183 (71.76)	161 (77.03)	66 (69.47)	28 (66.67)	40 (58.82)	9.174	0.057
	Yes	72 (28.24)	48 (22.97)	29 (30.53)	14 (33.33)	28 (41.18)		
Chronic obstructive pulmonary disease	NO	238 (93.33)	176 (84.21)	86 (90.53)	36 (85.71)	58 (85.29)	11.213	**0.024**
	Yes	17 (6.67)	33 (15.79)	9 (9.47)	6 (14.29)	10 (14.71)		
Diabetes	NO	173 (67.84)	141 (67.46)	61 (64.21)	28 (66.67)	46 (67.65)	Fisher	0.908
	Yes	76 (29.8)	59 (28.23)	29 (30.53)	13 (30.95)	21 (30.88)		
	With organ injuries	6 (2.35)	9 (4.31)	5 (5.26)	1 (2.38)	1 (1.47)		
Moderate/severe chronic kidney disease	NO	206 (80.78)	179 (85.65)	72 (75.79)	35 (83.33)	52 (76.47)	5.700	0.223
	Yes	49 (19.22)	30 (14.35)	23 (24.21)	7 (16.67)	16 (23.53)		
Solid tumors	NO	197 (77.25)	167 (79.9)	74 (77.89)	32 (76.19)	47 (69.12)	Fisher	0.236
	Yes	45 (17.65)	39 (18.66)	18 (18.95)	7 (16.67)	19 (27.94)		
	With multiple metastases	13 (5.1)	3 (1.44)	3 (3.16)	3 (7.14)	2 (2.94)		
Hypertension	NO	76 (29.8)	71 (33.97)	28 (29.47)	13 (30.95)	29 (42.65)	4.689	0.321
	Yes	179 (70.2)	138 (66.03)	67 (70.53)	29 (69.05)	39 (57.35)		
Coronary heart disease	NO	152 (59.61)	136 (65.07)	58 (61.05)	24 (57.14)	35 (51.47)	4.444	0.349
	Yes	103 (40.39)	73 (34.93)	37 (38.95)	18 (42.86)	33 (48.53)		

*The bold values mean P < 0.05*.

### Correlations of Infection With Age and Nutritional Status

Among all patients, the infection outcome was negatively correlated with age (*P* = 0.013). The level of albumin was negatively correlated with infection prognosis in all patients (*P* = 0.03) (Table 2), and negatively correlated with neutrophil count and CRP (*P* = 0.008, *P* < 0.001) (Tables 3, 4).

**Table 2:** Aging-related risk factors (age, nutrition, and comorbidities) on the outcome of pneumonia in all infected patients.

**Duration of infection**	**Coef**	***t***	***P***	**95%CI**	
Age (year)	−0.21	−2.490	**0.013**	−0.37	−0.04
Albumin (g/L)	−0.29	−2.180	**0.030**	−0.55	−0.03
aCCI	0.08	2.490	**0.013**	0.02	0.15
Myocardial infarction	0.24	1.040	0.298	−0.21	0.70
Peripheral vascular disease	−0.07	−0.320	0.749	−0.51	0.37
Cerebrovascular disease/TIA	−0.06	−0.410	0.685	−0.32	0.21
Hemiplegia	2.12	1.770	0.077	−0.23	4.47
Dementia/Alzheimer's disease	0.33	2.270	**0.024**	0.04	0.61
Chronic obstructive pulmonary disease/Asthma	0.45	2.210	**0.028**	0.05	0.85
Rheumatoid/connective tissue disease	−0.13	−0.280	0.778	−1.00	0.75
Peptic ulcer	0.07	0.090	0.925	−1.41	1.56
Diabetes	−0.02	−0.150	0.882	−0.25	0.22
Moderate/severe chronic kidney disease	0.28	1.660	0.098	−0.05	0.61
Liver disease	0.11	0.280	0.777	−0.66	0.88
Solid tumor	0.12	0.980	0.327	−0.12	0.36
Lymphoma	0.50	1.280	0.200	−0.27	1.27
Hypertension	−0.17	−1.230	0.218	−0.44	0.10
Coronary heart disease	0.11	0.800	0.424	−0.16	0.38

*The bold values mean P < 0.05*.

**Table 3:** Correlation of age-related risk factors (age, nutrition, and comorbidities) in Neutrophils count in all infected patients.

**Neutrophils count (10^**9**^/L)**	**Coef**	***t***	***P***	**95%CI**	
Age (year)	0.02	0.120	0.907	−0.38	0.43
Albumin (g/L)	−0.89	−2.650	**0.008**	−1.55	−0.23
aCCI	0.19	2.340	**0.020**	0.03	0.35
Myocardial infarction	0.69	1.190	0.236	−0.45	1.83
Peripheral vascular disease	0.49	0.870	0.386	−0.62	1.60
Cerebrovascular disease/TIA	−0.16	−0.460	0.644	−0.83	0.51
Hemiplegia	5.35	1.780	0.075	−0.55	11.25
Dementia/Alzheimer's disease	0.35	0.950	0.343	−0.37	1.06
Chronic obstructive pulmonary disease/Asthma	0.15	0.300	0.766	−0.85	1.16
Rheumatoid/connective tissue disease	0.83	0.750	0.456	−1.35	3.01
Peptic ulcer	1.74	0.920	0.358	−1.98	5.47
Diabetes	0.14	0.470	0.636	−0.45	0.73
Moderate/severe chronic kidney disease	0.00	0.000	0.998	−0.83	0.83
Liver disease	1.09	1.100	0.270	−0.85	3.03
Solid tumor	0.47	1.500	0.133	−0.14	1.07
Lymphoma	−1.28	−1.230	0.218	−3.31	0.76
Hypertension	0.72	2.060	**0.040**	0.03	1.40
Coronary heart disease	−0.11	−0.310	0.754	−0.78	0.57

*The bold values mean P < 0.05*.

**Table 4:** Correlation of age-related risk factors (age, nutrition, and comorbidities) in CRP in all infected patients.

**CRP**	**Coef**	***t***	***P***	**95%CI**	
Age (year)	−1.95	−0.650	0.518	−7.89	3.98
Albumin (g/L)	−16.73	−3.550	**<0.001**	−25.99	−7.47
aCCI	3.28	2.940	**0.003**	1.09	5.48
Myocardial infarction	−2.91	−0.340	0.737	−19.93	14.11
Peripheral vascular disease	−4.46	−0.610	0.539	−18.72	9.80
Cerebrovascular disease/TIA	5.90	1.220	0.223	−3.60	15.41
Hemiplegia	23.88	0.750	0.456	−38.99	86.75
Dementia/Alzheimer's disease	−1.62	−0.320	0.752	−11.71	8.46
Chronic obstructive pulmonary disease/Asthma	4.89	0.710	0.481	−8.74	18.52
Rheumatoid/connective tissue disease	10.17	0.670	0.505	−19.80	40.13
Peptic ulcer	29.41	0.930	0.355	−33.06	91.88
Diabetes	2.22	0.530	0.597	−6.01	10.44
Moderate/severe chronic kidney disease	−1.50	−0.250	0.802	−13.24	10.25
Liver disease	4.52	0.280	0.780	−27.31	36.35
Solid tumor	15.45	3.560	**<0.001**	6.92	23.97
Lymphoma	25.45	1.850	0.065	−1.61	52.51
Hypertension	3.01	0.610	0.543	−6.71	12.72
Coronary heart disease	−6.87	−1.440	0.151	−16.26	2.52

*The bold values mean P < 0.05*.

### Correlations of Infection With aCCI and Underlying Diseases

Among all patients, the infection outcome was negatively correlated with aCCI (*P* = 0.013) (Table 2). aCCI was positively correlated with neutrophils count and CRP (*P* = 0.02, *P* = 0.003) (Tables 3, 4).

Prognosis varies among patients with different underlying diseases. Among all patients, the severity of infection outcome was positively correlated with dementia/Alzheimer's disease (*P* = 0.024), chronic obstructive pulmonary disease/asthma (*P* = 0.028) (Table 2).

**Figure 1:**
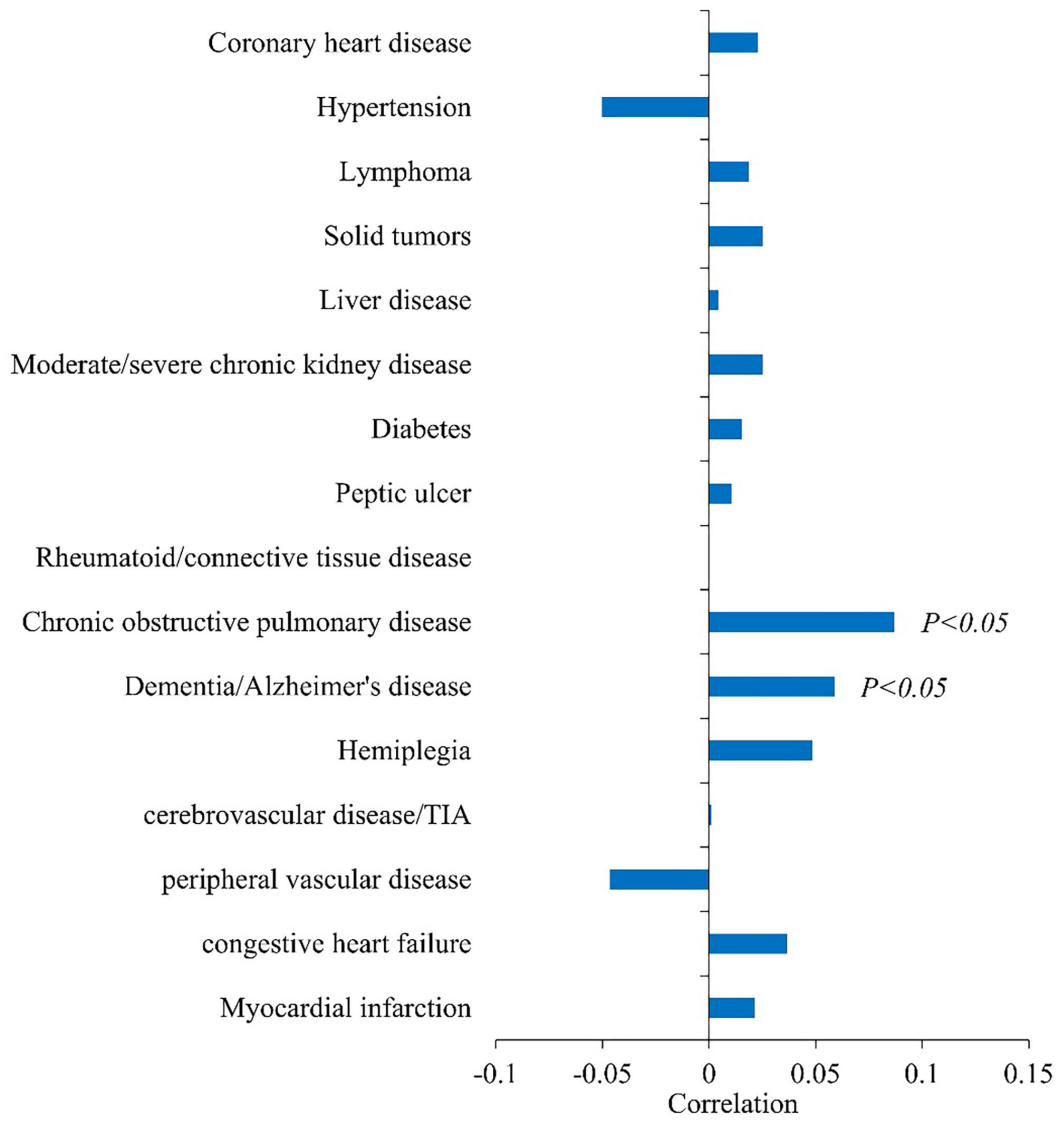
Correlation analysis between basic diseases and infection outcomes (Spearman's correlation coefficient in abscess).

Figure 1 shows the correlation between underlying disease and infection outcome. Chronic obstructive pulmonary disease (correlation coefficient = 0.0868, *P* < 0.05) and dementia/Alzheimer's disease (correlation coefficient = 0.0589, *P* < 0.05) were positively associated with infection outcome.

### Associations Between Nutritional Status, Infection Level, and Pneumonia

**Figure 2:**
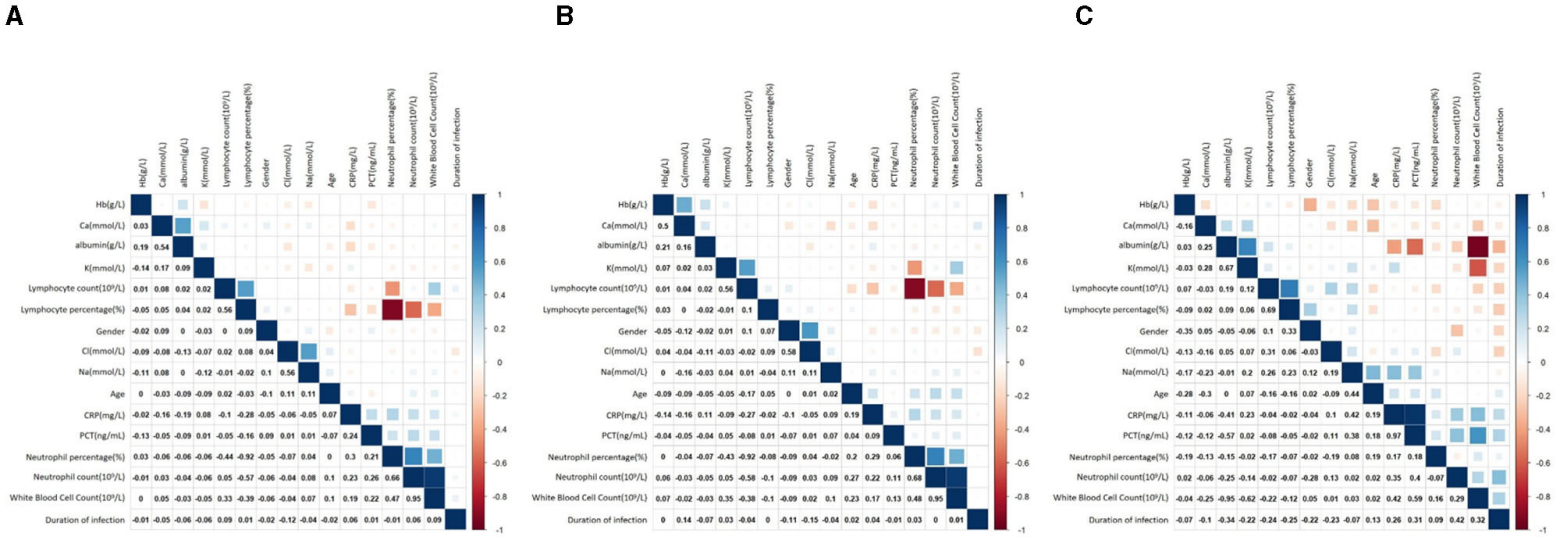
Heat map of correlation analysis. **(A)** Associations between nutritional status, infection levels and pneumonia in all infected patients. **(B)** Associations between nutritional status, infection levels and pneumonia in HAP. **(C)** Associations between nutritional status, infection levels and pneumonia in CAP.

The correlation coefficient matrix was shown by the heat map (Figure 2). The red region denotes a high positive correlation, and the blue region indicates a negative correlation. In all infected patients, HAP, and CAP groups, there was an obvious correlation between nutrition-related indicators and inflammatory indicators.

**Figure 3:**
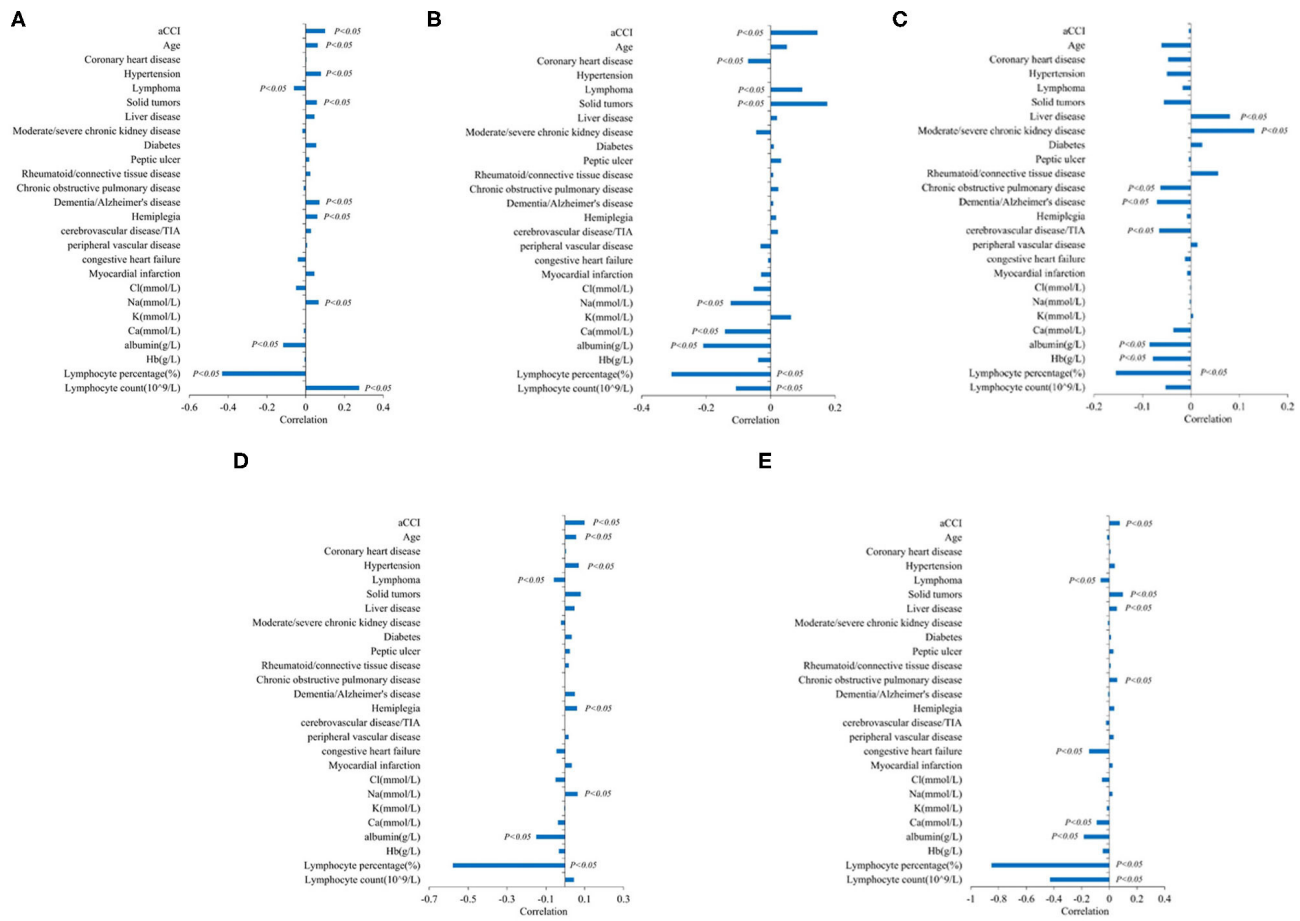
Correlation analysis of aging-related risk factors and infection. **(A)** Correlation analysis of aging-related risk factors and white blood cell level. **(B)** Correlation analysis of aging-related risk factors and CRP. **(C)** Correlation analysis of aging-related risk factors and PCT. **(D)** Correlation analysis of aging-related risk factors and neutrophils count(10^∧^9/L). **(E)** Correlation analysis of aging-related risk factors and neutrophils percentage (%).

To understand the correlations between the variables and infection, we used the correlation coefficient considering the whole population of interest (Figure 3).

In all infected patients, age was positively correlated with leukocyte level and neutrophil count (*P* < 0.05), but had no statistical significance with CRP, PCT, and neutrophil percentage.

In nutritional markers, albumin was negatively correlated with all inflammatory markers (CRP, PCT, leucocyte level, neutrophil percentage, and neutrophil count). Different types of electrolytes had different associations with specific inflammatory markers, especially Na and Ca. Na was negatively correlated with CRP and positively correlated with WBC and neutrophil count. Ca was negatively correlated with CRP and neutrophil percentage, and there was no statistically significant relationship between other electrolytes and inflammatory indicators (*P* < 0.05).

Indicators of the severity of comorbidity: aCCI was positively correlated with CRP, WBC, neutrophil count, and percentage of neutrophils (*P* < 0.05).

### Predictive Model for the Infection Outcomes

**Figure 4:**
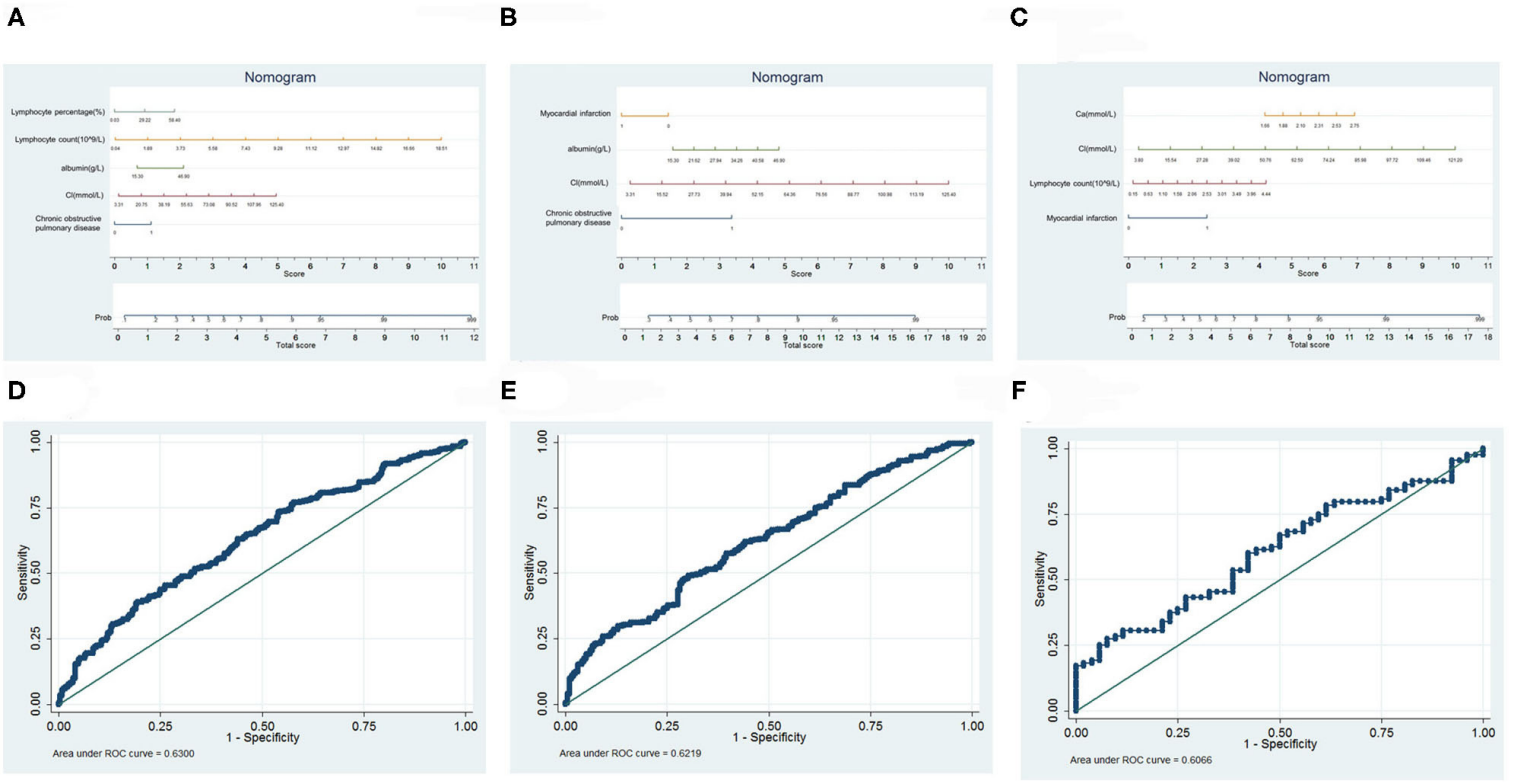
Prediction model of infection outcome. **(A)** Nomogram of all infected patients. **(B)** Nomogram of HAP. **(C)** Nomogram of CAP. **(D)** ROC curves of all infected patients. **(E)** ROC curves of HAP. **(F)** ROC curves of CAP.

Based on logistic, dichotomous and sequentially screened analyses, a nomogram incorporating the seven risk factors was built for predicting infection outcome (Figure 4). Discrimination was performed by ROC curves.

Of all those infected, a total score was calculated with the use of lymphocyte percentage, lymphocyte count, albumin, Cl, and chronic obstructive pulmonary disease. The area under the curve (AUC) was 0.6300. In the HAP group, we selected myocardial infarction, albumin, Cl, and chronic obstructive pulmonary disease as risk factors that collaboratively achieved an AUC of 0.6219. In the CAP group, we used Ca, Cl, lymphocyte count and myocardial infarction to calculate the total score, achieving an AUC of 0.6066.

## Discussion

According to the World Health Organization, 450 million people, or 7 percent of the global population, are diagnosed with pneumonia each year, and about 4 million people die from it (21). The elderly are more likely to suffer from this disease because of their declined organ function, aging immune system, basic diseases, poor nutrition and other abnormalities. In addition, compared with that in normal adults, pneumonia in the elderly brings higher medical costs and worse clinical outcomes. Severe cases may demonstrate multiple organ dysfunction syndrome (MODS), respiratory and circulatory failure, even death (22, 23). Pneumonia has become one of the leading causes of death in the elderly (24). This study found that pneumonia in the elderly was correlated with nutrition status, basic diseases and age, providing a reference for clinical comprehensive treatment.

Some elderly patients with pneumonia show signs of loss of appetite, listlessness, etc. Reduced nutrient intake leads to poor nutritional status and aggravates infection in elderly patients (25). Poor nutritional status damages respiratory function, thus impairing immune function and increasing pneumonia (26, 27). Adequate nutrition can enhance respiratory muscle function and immune defense (28, 29). Therefore, nutritional intervention can control disease progression and improve respiratory function, thus reducing morbidity and mortality (30), hospitalization costs, and re-hospitalizations (31, 32). Albumin level below 3.5 mg/dL suggests a poor nutritional status and survival (33). There is a correlation between pneumonia and albumin level in elderly patients. Therefore, albumin level <3.5 mg/dL is a risk factor for the occurrence and poor prognosis of pneumonia in elderly patients.

Poor nutritional status is a common comorbidity of the elderly, and it interacts with other basic diseases. In this study, elderly patients with pneumonia were further divided into HAP and CAP groups. It was found that for both CAP and HAP patients, nutritional status was a risk factor for the occurrence and prognosis of pneumonia, compared with other underlying diseases. Common underlying diseases have an impact on nutrition include diabetes, COPD, cerebrovascular disease, hemiplegia, dementia, peptic ulcer, moderately severe chronic kidney disease, liver disease (34). Nutritional status differs across regions (35, 36). In addition to albumin and some others biochemical indicators, the Mini Nutritional Assessment Table (MNA) and MNA-SF (Short Form MNA) are fast and reliable methods for assessing the nutritional status of the elderly (37–42). Nutritional screening and targeted nutrition education should be carried out to reduce the incidence of poor nutritional status and pneumonia in the elderly.

Multiple underlying diseases also predispose the elderly to pneumonia, such as cerebral infarction, cerebral hemorrhage and other cerebrovascular diseases and left swallowing dysfunction. Esophageal tracheal fistula can also cause aspiration pneumonia, which is the most common pneumonia in the elderly (43). Patients with sequelae of cerebral infarction (such as hemiplegia) are prone to pneumonia, because of mobility difficulties and long-term bed rest. Patients with COPD have a higher susceptibility to respiratory infections (44, 45). In this study, the course of pneumonia was significantly prolonged in patients with COPD, dementia, cerebrovascular disease, and hemiplegia. Similarly, tumor was also identified as a risk factor for pneumonia in the elderly patients. It may be due to the decline of immunity after the use of hormones, immunosuppressants, or chemotherapy (46, 47).

At the end of the study, a model to predict the duration of pneumonia in elderly patients based on common nutritional indicators and basic diseases was obtained, which is feasible, simple and convenient, and suitable for the evaluation of clinical conditions of ordinary patients. The model can predict the course of disease and adjust the diagnosis and treatment plan in time to avoid increasing the burden of disease. However, due to the small number of model parameters, the guiding significance to clinical needs to be further confirmed and strengthened.

This study is a retrospective study and has its limitations. A correlation analysis was performed only for the available outcomes in elderly patients with pneumonia during hospitalization. In the future, we will design and do more rigorous and scientific prospective study to investigate more relevant nutritional indicators, such as nutritional assessment scale, index of body examination and the clinical biochemistry, bioelectrical impedance analysis (BIA), and conduct long-term follow-up of patients' nutritional status, such as retreatment or readmission after discharge. Thus, we believe that our further results can provide a more intuitive and reliable reference for nutritional intervention of elderly patients with pneumonia.

## Conclusions

Both for HAP and CAP patients, advanced age and comorbidities are risk factors for the occurrence and outcome of pneumonia in the elderly. Poor nutritional status and COPD have significant effects on the severity and course of pneumonia in the elderly, and can be used as risk factors to predict the risk and prognosis of pneumonia. The disease should be managed with anti-infection treatment, but also efforts to prevent poor nutritional status and comorbidities.

## Data Availability Statement

The original contributions presented in the study are included in the article/supplementary material, further inquiries can be directed to the corresponding authors.

## Ethics Statement

The studies involving human participants were reviewed and approved by the Ethics Committee of the First Affiliated Hospital of Nanjing Medical University. The patients/participants provided their written informed consent to participate in this study.

## Author Contributions

BC designed the study and statistical analysis, interpreted the data, and drafted the manuscript. WL and YC collected the epidemiological and clinical data, interpreted the data, and drafted the manuscript. QS, ML, HZ, and WZ contributed to the data analysis and interpretation and revised the manuscript. JW and ZP designed the study, interpreted the data, and revised the manuscript. All authors approved the final version of the manuscript.

## Funding

This study was supported by grants from the National Key R&D Program of China (Nos. 2018YFC2002100 and 2018YFC2002102), the National Natural Science Foundation of China (No. 81871115), the Cadre Health Care Research Project of Jiangsu Province (No. BJ20018), the Natural Science Research Project of Colleges and Universities in Jiangsu Province (No. 20KJB320002), the Outstanding Young and Middle-aged Talents Support Program of the First Affiliated Hospital with Nanjing Medical University, the Six Talent Peaks Project in Jiangsu Province (No. 2018-WSN-003), the Jiangsu Province's Youth Medical Talents Program (No. QNRC2016593), and Natural Science Foundation of Jiangsu Province (No. BK20211377).

## Conflict of Interest

The authors declare that the research was conducted in the absence of any commercial or financial relationships that could be construed as a potential conflict of interest.

## Publisher's Note

All claims expressed in this article are solely those of the authors and do not necessarily represent those of their affiliated organizations, or those of the publisher, the editors and the reviewers. Any product that may be evaluated in this article, or claim that may be made by its manufacturer, is not guaranteed or endorsed by the publisher.
